# Exploring health care providers’ engagement in prevention and management of multidrug resistant Tuberculosis and its factors in Hadiya Zone health care facilities: qualitative study

**DOI:** 10.1186/s12913-024-10911-6

**Published:** 2024-04-27

**Authors:** Bereket Aberham Lajore, Yitagesu Habtu Aweke, Samuel Yohannes Ayanto, Menen Ayele

**Affiliations:** 1 Department of Family Health, Hossana College of health sciences, Hossana, Ethiopia; 2 Department of Health informatics, Hossana College of Health Sciences, Hossana, Ethiopia; 3 Department of Midwifery, Hossana College of Health Sciences, Hossana, Ethiopia; 4 Department of Clinical Nursing, Hossana College of Health Sciences, Hossana, Ethiopia; 5 Present Address: Hossana College of Health Sciences, Hosanna, SNNPR Ethiopia; 6 Present Address: College of Health Sciences, School of Public Health, Addis Ababa University, Addis Ababa, Ethiopia; 7 Present Address: College of Health Sciences, Institute of Public Health, Department of -Population and Family Health, Jimma University, Jimma, Ethiopia

**Keywords:** DOTS, DS-TB, Engagement, Healthcare providers, MDR-TB, XDR-TB

## Abstract

**Background:**

Engagement of healthcare providers is one of the World Health Organization strategies devised for prevention and provision of patient centered care for multidrug resistant tuberculosis. The need for current research question rose because of the gaps in evidence on health professional’s engagement and its factors in multidrug resistant tuberculosis service delivery as per the protocol in the prevention and management of multidrug resistant tuberculosis.

**Purpose:**

The purpose of this study was to explore the level of health care providers’ engagement in multidrug resistant tuberculosis prevention and management and influencing factors in Hadiya Zone health facilities, Southern Ethiopia.

**Methods:**

Descriptive phenomenological qualitative study design was employed between 02 May and 09 May, 2019. We conducted a key informant interview and focus group discussions using purposely selected healthcare experts working as directly observed treatment short course providers in multidrug resistant tuberculosis treatment initiation centers, program managers, and focal persons. Verbatim transcripts were translated to English and exported to open code 4.02 for line-by-line coding and categorization of meanings into same emergent themes. Thematic analysis was conducted based on predefined themes for multidrug resistant tuberculosis prevention and management and core findings under each theme were supported by domain summaries in our final interpretation of the results. To maintain the rigors, Lincoln and Guba’s parallel quality criteria of trustworthiness was used particularly, credibility, dependability, transferability, confirmability and reflexivity.

**Results:**

Total of 26 service providers, program managers, and focal persons were participated through four focus group discussion and five key informant interviews. The study explored factors for engagement of health care providers in the prevention and management of multidrug resistant tuberculosis in five emergent themes such as patients’ causes, perceived susceptibility, seeking support, professional incompetence and poor linkage of the health care facilities. Our findings also suggest that service providers require additional training, particularly in programmatic management of drug-resistant tuberculosis.

**Conclusion:**

The study explored five emergent themes: patient’s underlying causes, seeking support, perceived susceptibility, professionals’ incompetence and health facilities poor linkage. Community awareness creation to avoid fear of discrimination through provision of support for those with multidrug resistant tuberculosis is expected from health care providers using social behavioral change communication strategies. Furthermore, program managers need to follow the recommendations of World Health Organization for engaging healthcare professionals in the prevention and management of multidrug resistant tuberculosis and cascade trainings in clinical programmatic management of the disease for healthcare professionals.

**Supplementary Information:**

The online version contains supplementary material available at 10.1186/s12913-024-10911-6.

## Introduction

Mycobacterium tuberculosis, the infectious agent that causes multi-drug resistant tuberculosis (MDR-TB), is resistant to at least rifampicin and isoniazid. Direct infection can cause the disease to spread, or it can develop secondary to improper management of tuberculosis among drug susceptible tuberculosis cases and associated poor adherence [1].

Multidrug-resistant strains of mycobacterium tuberculosis have recently emerged, which makes achieving “End TB Strategy” more difficult [2]. Multi drug resistant tuberculosis (MDR-TB) has been found to increasingly pose a serious threat to global and Ethiopian public health sector. Despite the fact that a number of risk factors for MDR-TB have been identified through various research designs, the epidemiology of this disease is complex, contextual, and multifaceted [1]. Quantitative studies demonstrate that prior treatment history [3–7], interrupted drug supply [8], inappropriate treatments and poor patient compliance [3, 7, 9], poor quality directly observed treatment short course (DOTS), poor treatment adherence [10], age [5], and malnutrition [11] were factors associated with multi drug resistant TB.

Globally, an estimated 20% of previously treated cases and 3.3% of new cases are thought to have MDR-TB; these levels have essentially not changed in recent years. Globally, 160,684 cases of multidrug-resistant TB and rifampicin-resistant TB (MDR/RR-TB) were notified in 2017, and 139,114 cases were enrolled into treatment in 2017 [12]. A systematic review in Ethiopia reported 2% prevalence of MDR-TB [3] that is higher than what is observed in Sub-Saharan Africa, 1.5% [13]. The prevalence of MDR-TB, according to the national drug-resistant tuberculosis (DR-TB) sentinel report, was 2.3% among newly diagnosed cases of TB and 17.8% among cases of TB who had already received treatment,. This suggests a rising trend in the prevalence of TB drug resistance compared to the results of the initial drug-resistant TB survey carried out in Ethiopia from 2003 to 2005 [14].

Ethiopia has placed strategies into place that emphasize political commitment, case finding, appropriate treatment, a continuous supply of second-line anti-TB medications of high quality, and a recording system. Due to other competing health priorities, the nation is having difficulty accelerating the scale-up of the detection, enrollment and treatment of drug-resistant TB patients [15, 16]. To address these issues, the nation switched from a hospital-based to a clinic-based ambulatory model of care, which has allowed MDR-TB services to quickly decentralize and become more accessible. Accordingly, the nation has set up health facilities to act as either treatment initiating centers (TIC) or treatment follow-up centers (TFC) or both for improved referral and communication methods [15].

One of the key components of the “End TB strategy” is engagement of health care professionals in the prevention and management of multidrug resistant tuberculosis [17]. Inadequate engagement of healthcare providers is one aspect of the healthcare system that negatively influences MDR-TB prevention and control efforts [17]. This may be manifested in a number of ways, including inadequate understanding of drug-resistant tuberculosis, improper case identification, failure to initiate treatment again, placement of the wrong regimens, improper management of side effects and poor infection prevention [1]. These contributing factors are currently being observed in Ethiopia [18], Nigeria [7, 19, 20] and other countries [21, 22]. According to a study conducted in Ethiopia, MDR-TB was linked to drug side effects from first-line treatments, being not directly observed, stopping treatment for at least a day, and retreating with a category II regimen [17].

This may be the result of a synergy between previously investigated and other contextual factors that have not yet been fully explored, such as professional engagement, beliefs, and poor preventive practices. The engagement of health professionals in MDR-TB prevention and control is assessed using a number of composite indicators. Health professionals may interact primarily inside the healthcare facilities. Typically, they play a significant role in connecting healthcare services with neighborhood-based activities [17]. One of the main research areas that have not sufficiently addressed is evidence indicating the status of healthcare professionals’ engagement and contextual factors in MDR-TB prevention and management.

It is increasingly urgent to identify additional and existing factors operating in a particular context that contribute to the development of the disease in light of the epidemic of drug resistance, including multi-drug resistance (MDR-TB) and extensively drug resistant TB (XDR-TB) in both new and previously treated cases of the disease [23]. In order to develop and implement control measures, it is therefore essential to operationally identify a number of contextual factors operating at the individual, community, and health system level.

Therefore, the overall purpose of this study was to explore the level of engagement of health care providers and contextual factors hindering/enabling the prevention and provision of patient-centered care for MDR-TB in health facilities, DOTS services centers and MDR-TB treatment initiation center [TIC], in Hadiya Zone, Southern Ethiopia.

## Methods

### Qualitative approach and research paradigm

Descriptive phenomenological qualitative study design was employed to explore factors influencing engagement of health professionals in MDR-TB prevention and management and thematic technique was employed for the analysis of the data.

### Researchers’ characteristics and reflexivity

Three Principal investigators conducted this study. Two of them had Masters of public health in Epidemiology and Reproductive health and PhD candidates and the third one had Bachelor’s degree in public health with clinical experience in the area of Tuberculosis prevention and management and MPH in Biostatistics. The principal investigators have research experience with published articles in different reputable journals. There were no prior contacts between researchers and participants before the study whereas researchers have built positive rapport with study participants during data collection to foster open communication and trust and had no any assumptions and presuppositions about the research topic and result.

### Context/ study setting and period

The study was conducted between 2 and 9 May, 2019 in Hadiya Zone with more than 1.7 million people residing in the Zone. There are 300 health posts, 63 health centers, 3 functional primary hospitals and 1 comprehensive specialized hospital in the Zone. Also, there are more than 350 private clinics and 1 private hospital in the Zone. All of the public health facilities and some private health facilities provide directly observed short course treatment (DOTS) service for tuberculosis patients. There are more than eight treatment initiation centers (TICs) for MDR-TB patients in Hadiya Zone. MDR-TB (Multidrug-resistant tuberculosis) treatment initiation centers are specialized facilities that provide comprehensive care, diagnosis and treatment initiation, psychosocial support, and follow up services to individuals with MDR-TB. The linkage between MDR-TB treatment initiation centers and other healthcare facilities lies in the coordination of care, referral pathways, and collaboration to ensure comprehensive and integrated care for individuals with MDR-TB. Overall, healthcare providers play a crucial role in the management of MDR-TB by providing specialized care, ensuring treatment adherence, monitoring progress and outcomes, and supporting individuals in achieving successful treatment outcomes and improved health.

### Units of study and sampling strategy

Our study participants were health care professionals working in MDR-TB TICs in both private and public health facilities, and providing DOTS services, MDR-TB program leaders in treatment initiation centers, as well as TB focal persons, disease prevention and health promotion focal person, and project partners from district health offices. The study involved four focus group discussion (FGDs) and five key informants’ interview (KII) with a total of 26 participants to gather the necessary information. Expert purposive sampling technique was employed and sample size was determined based on the saturation of idea required during data collection process.

### Data collection methods and instruments

Focus group discussion and face to face key informants’ interviews were employed to collect the data. We conducted a total of four FGD and five key informants’ interviews with participants chosen from DOTS providing health facilities and MDR-TB program leaders in treatment initiation centers, as well as TB focal persons and project partners from district health offices and disease prevention and health promotion focal person. One of the FGDs was conducted among health professionals from the public MDR-TB treatment initiation centers. Three FGDs were conducted among disease prevention and health promotion focal persons, TB focal persons and DOTS providers in public health facilities (health centers).

An observation checklist was developed to assess the general infection prevention and control measures used by specific healthcare facilities in the study area. We used unstructured FGD guide, key informant interview guide, observation checklist and audio recorders to collect primary data and it was collected using local language called Amharic. Prior to data collection, three people who are not among principal investigators with at least a master’s degree in public health and prior experience with qualitative research were trained by principal investigators. Three of them acts as a tape recorder, a moderator, and as a note taker alternatively. The length of FGD ranged from 58 to 82 min and that of key informants’ interview lasted from 38 to 56 min.

### Data processing and data analysis

Memos were written immediately after interviews followed by initial analysis. Transcription of audio records was performed by principal investigators. The audio recordings and notes were refined, cleaned and matched at the end of each data collection day to check for inconsistencies, correct errors, and modify the procedures in response to evolving study findings for subsequent data collection. Transcribed interviews, memos, and notes from investigator’s observation were translated to English and imported to Open Code 4.02 [2] for line by line coding of data, and categorizing important codes (sub theming). The pre-defined themes for MDR-TB prevention and control engagement were used to thematize the line-by-line codes, categories, and meanings using thematic analysis. Finally, the phenomenon being studied was explained by emerging categories and themes. Explanations in themes were substantiated by participants’ direct quotations when necessary.

### Trustworthiness

Phone calls and face to face briefing were requested from study participants when some expressions in the audio seems confusing while transcripts were performed. To ensure the credibility of the study, prolonged engagement was conducted, including peer debriefing with colleagues of similar status during data analysis and inviting available study participants to review findings to ensure as it is in line with their view or not. Memos of interviews and observation were crosschecked while investigator was transcribing to ensure credibility of data as well as to triangulate investigator’s categorizing and theming procedures. For transferability, clear outlines of research design and processes were provided, along with a detailed study context for reader judgment. Dependability was ensured through careful recording and transcription of verbal and non-verbal data, and to minimize personal bias, scientific procedures were followed in all research stages. Conformability was maintained by conducting data transcription, translation, and interpretation using scientific methods. Researchers did all the best to show a range of realities, fairly and faithfully. Finally, an expert was invited to put sample of codes and categories to emerged corresponding categories and themes respectively.

## Results

### Demographic characteristics of study participants

Four focus group discussions and five key informants’ interviews were conducted successfully. There were 26 participants in four focus group discussions, and key informants’ interview. Ages of participants ranges from 20 to 50 years with an average age of 33.4 ± 6.24 SD years. Participants have five to ten years of professional experience with DOTS services (Table 1).

**Table 1:** Socio demographic details of study participants from different health facilities working in MDR-TB prevention and management, 2019

Demographic characteristics of study participants (*n* = 26)		
Variables	Number	Percent
**Age group**		
20–30	10	38.5
31–40	13	50.0
41–50	3	11.5
**Sex**		
Male	21	80.8
Female	5	19.2
**Marital Status**		
Married	18	69.2
Single	5	19.2
Separated	3	11.5
**Educational Status**		
Diploma ( Level IV or 10 + 3)	16	61.5
BSc degree	8	30.8
Second degree (MPH)	2	7.7
**Profession**		
Clinical Nursing	18	69.2
Public health officer	6	23.1
Public health specialist (MPH)	2	7.7
Professional experience (years )	Mean ± SD	
Average years of experience	7.93 ± 3.84	
Average years of experience in DOTS/MDR-TB-TICs	2.97 ± 2.77	

### Emergent themes and subthemes

**Fig. 1:**
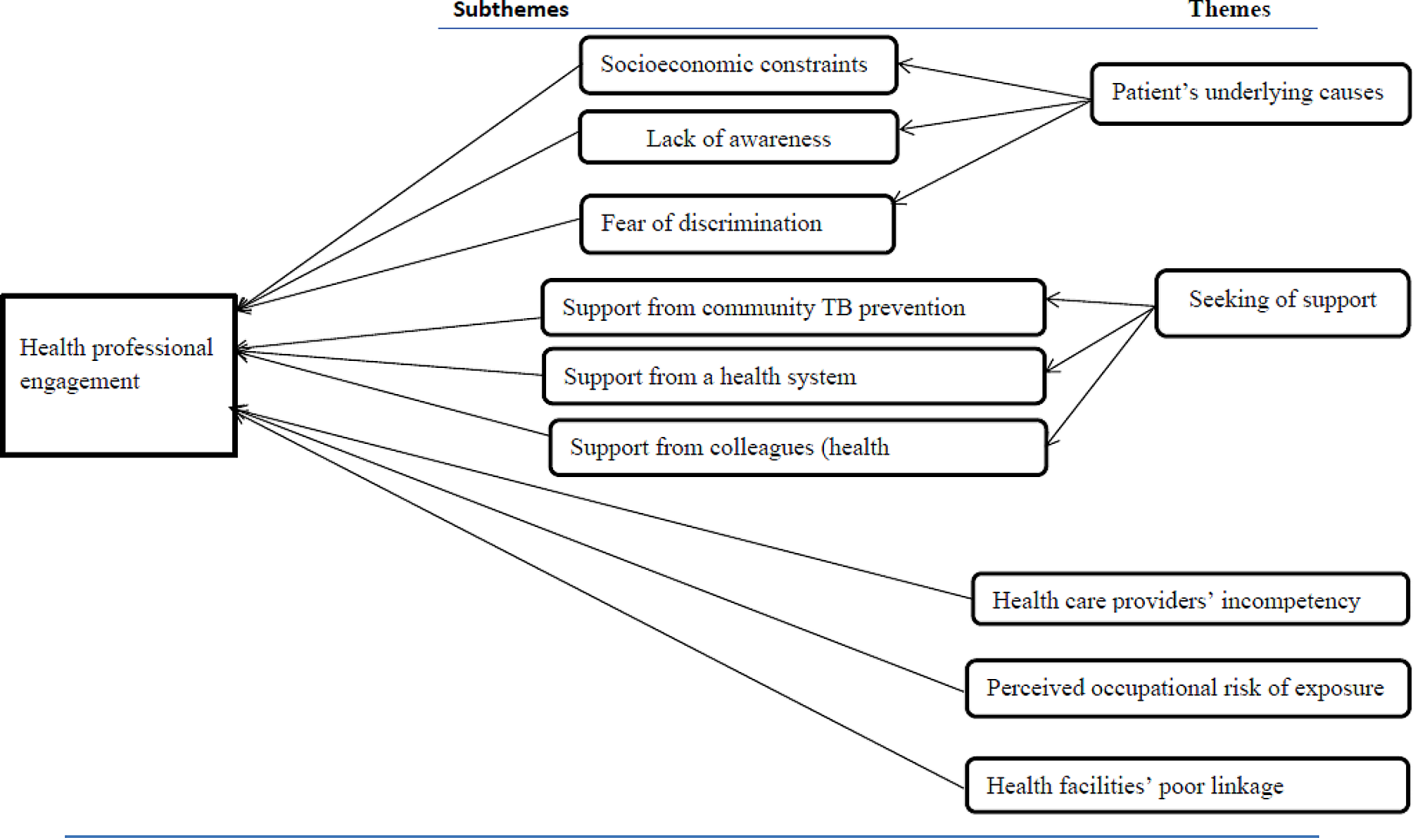
Themes and subthemes emerged from the analysis of health professionals’ engagement in MDR-TB prevention and management study in Hadiya zone’s health facilities, 2019

The study explored how health care providers’ engagement in MDR-TB prevention and management was influenced. The investigation uncovered five major themes. These themes were the patient’s underlying causes, seeking support, perceived susceptibility, healthcare providers’ incompetence, and poor linkage between health facilities. Weak community TB prevention, health system support, and support from colleagues were identified subthemes in the search for help by health professionals whereas socioeconomic constraints, lack of awareness, and fear of discrimination were subthemes under patients underlying factors (Fig. 1).

#### The patient’s underlying causes

This revealed why TB/MDR-TB treatment providers believe health professionals are unable to provide standard MDR-TB services. The subthemes include TB/MDR-TB awareness, fear of discrimination, and patients’ socioeconomic constraints.

##### Socioeconomic constraints

According to our research, the majority of healthcare professionals who provided directly observed short-course treatment services mentioned socioeconomic constraints as barriers to engage per standard and provide MDR-TB prevention and management service. More than half of the participants stated that patients’ primary reasons include lack of money for house rental close to the treatment centers, inability to afford food and other expenses, and financial constraints to cover transportation costs.

In addition to this, patients might have additional responsibilities to provide food and cover other costs for their families’ need. The majority of health care professionals thought that these restrictions led to their poor engagement in MDR-TB prevention and management. One of the focus groups’ discussants provided description of the scenario in the following way:

*“…. I have many conversations with my TB/MDR-TB patients. They fail to complete DOTS or treatment intensive care primarily as a result of the requirement of prolonged family separation. They might provide most of the family needs, including food and other expenses”* (FGD-P01).

##### Lack of awareness about MDR-TB

This subtheme explains how MDR-TB patients’ knowledge of the illness can make it more difficult for health professionals to provide DOTS or TICs services. The majority of DOTS providers stated that few TB or MDR-TB patients were aware of how MDR-TB spreads, how it is treated, and how much medication is required. Additionally, despite the fact that they had been educated for the disease, majority of patients did not want to stop contact with their families or caregivers. A health care provider stated,

“…. *I provided health education for MDR-TB patients on how the disease is transmitted and how they should care for their family members. They don’t care; however, give a damn about their families*.” (FGD-P05).

Some healthcare professionals reported that some patients thought that MDR-TB could not be cured by modern medication. One medical professional described the circumstance as follows:

“…. *I noticed an MDR-TB patient who was unwilling to be screened. He concluded that modern medication is not effective and he went to spiritual and traditional healers”* (FGD-P02).

As a result, almost all participants agreed on the extent to which patient knowledge of TB and MDR-TB can influence a provider’s engagement to MDR-TB services. The majority suggested that in order to improve treatment outcomes and preventive measures, the media, community leaders, health development armies, one-to-five networks, non-governmental organizations, treatment supporters, and other bodies with access to information need to put a lot of efforts.

##### Fear of discrimination

According to our research, about a quarter of healthcare professionals recognized that patients’ fear of discrimination prevents them from offering MDR-TB patients the DOTS services they need, including counseling index cases and tracing contact histories.

HEWs, HDAs, and 1-to-5 network members allegedly failed to monitor and counsel the index cases after their immediate return to their homes, according to the opinions from eight out of twenty-six healthcare professionals. The patients began to engage in routine social and political activities with neighbors while hiding their disease status. A healthcare professional described this situation as follows:

“…. *I understood from my MDR-TB patient’s words that he kept to himself and avoided social interaction. He made this decision as a result of stigmatization by locals, including health extension workers. As a result, the patient can’t attend social gatherings.*…. *In addition, medical professionals exclude MDR-TB patients due to fear of exposures. As a result, patients are unwilling to undergo early screening”* (FGD-P04).

#### Professionals’ perceived risk of occupational exposure

This theme highlights the anxiety that healthcare workers experience because of MDR-TB exposure when providing patient care. Our research shows that the majority of health professionals viewed participation as “taking coupons of death.” They believed that regardless of how and where they engaged in most healthcare facilities, the risk of exposure would remain the same. According to our discussion and interview, lack of health facility’s readiness takes paramount shares for the providers’ risk of exposures and their susceptibility.

According to the opinion from the majority of FGD discussants and in-depth interviewees, participants’ self-judgment score and our observation, the majority of healthcare facilities that offer DOTS for DS-TB and MDR-TB did not create or uphold standards in infection prevention in the way that could promote better engagement. These include poor maintenance of care facilities, lack of personal protective equipment, unsuitable facility design for service provision, lack of patient knowledge regarding the method of MDR-TB transmission, and lack of dedication on the part of health care staff.

As one of our key informant interviewees [District Disease Prevention Head], described health professionals’ low engagement has been due to fear of perceived susceptibility. He shared with us what he learned from a community forum he moderated.

Community forum participant stated that “… *There was a moment a health professional run-away from the TB unit when MDR-TB patient arrived. At least they must provide the necessary service, even though they are not willing to demonstrate respectful, compassionate, or caring attitude to MDR-TB patients”* (KII-P01). *Besides*, one of the FGD discussants described the circumstance as follows:

“…. *Emm…. Because most health facilities or MDR-TB TIC are not standardized, I am concerned about the risk of transmission. They are crammed together and poor ventilation is evident as well as their configuration is improper. Other medical services are causing the TICs to become overcrowded. Most patients and some medical professionals are unconcerned with disease prevention*” (FGD-P19).

Participants’ general fear of susceptibility may be a normal psychological reaction and may serve as a motivation for taking preventative actions. However, almost all participants were concerned that the main reasons for their fear were brought up by the improper application of programmatic management and MDR-TB treatment standards and infection prevention protocols in healthcare facilities.

### Health care providers’ incompetence

This theme illustrates how professionalism and dedication impact participation in MDR-TB prevention and management. The use of DS-TB prevention and management by health professionals was also taken into account because it is a major factor in the development of MDR-TB. This theme includes the participants’ perspectives towards other healthcare workers involved in and connected to MDR-TB.

Nearly all of the participants were aware of the causes and danger signs of MDR-TB. The majority of the defined participants fit to the current guidelines. However, participants in focus groups and key informant interviews have brought up shortcomings in MDR-TB service delivery practice and attitude. We looked at gaps among healthcare professionals’ knowledge, how they use the national recommendations for programmatic management and prevention of MDR-TB, prevent infections, take part in community MDR-TB screenings, and collaborate with other healthcare professionals for better engagement.

More than half of the participants voiced concerns about their attitudes and skill sets when using MDR-TB prevention and management guideline. When asked about his prior experiences, one of the focus group participants said:

*“…. Ok, let me tell you my experience, I was new before I attended a training on MDR-TB. I was unfamiliar with the MDR-TB definition given in the recommendations. When I was hired, the health center’s director assigned me in the TB unit. I faced difficulties until I received training”* (FGD-P24). *Furthermore*, one of the key informant interview participants shared a story:

*“…. In my experience, the majority of newly graduated health professionals lack the required skill. I propose that pre-service education curricula to include TB/MDR-TB prevention and management guideline trainings”* (KII-P01).

The majority of participants mentioned the skill gap that was seen among health extension workers and laboratory technicians in the majority of healthcare facilities. Some of the participants in the in-depth interviews and FGD described the gaps as follows:

*“…. According to repeated quality assurance feedbacks, there are many discordant cases in our [*District TB Focal Person*] case. Laboratory technicians who received a discrepant result* (KII-P01) *are not given training which is augmented by shared story from FGD discussants, “According to the quality assurance system, laboratory technicians lack skill and inconsistent results are typical necessitating training for newly joining laboratory technicians”* (FGD-P20).

Through our discussions, we explored the level of DOTS providers’ adherence to the current TB/MDR-TB guideline. As a result, the majority of participants pointed out ineffective anti-TB management and follow-up care. One of the participants remembered her practical experience as follows:

*“…. In my experience, the majority of health professionals fail to inform patients about the drug’s side effects, follow-up procedures, and other techniques for managing the burden of treatment. Only the anti-TB drug is provided, and the patient is left alone. The national treatment recommendation is not properly implemented by them”* (FGD-P04).

Many barriers have been cited as reasons that might have hindered competencies for better engagement of health professionals. Training shortage is one of the major reasons mentioned by many of the study participants. One of discussants from private health facility described the problem as

“…. *We are incompetent, in my opinion. Considering that we don’t attend update trainings. Many patients who were diagnosed negative at private medical facilities turned out to be positive, and vice versa which would be risky for drug resistance”* (FGD-P14) which was supported by idea from a participant in our in-depth interview: *“…. We [Program managers] are running short of training for our health care providers at different health centers and revealed that four out of every five healthcare professionals who work in various health centers are unaware of the TB/MDR-TB new guideline”* (KII-P02).

#### Seeking support

This theme focuses on the significance and effects of workplace support in the engagement of MDR-TB prevention and control. This also explains the enabling and impeding elements in the engagement condition of health professionals. Three elements make up the theme: coworkers (other health professionals) in the workplace, support from community TB prevention actors, and a healthcare system.

##### Support from community TB prevention actors

This subtheme includes the assistance provided to study participants by important parties such as community leaders, the health development army, and other stakeholders who were involved in a community-based TB case notification, treatment adherence, and improved patient outcomes.

Many of the study participants reported that health extension workers have been poorly participating in MDR-TB and TB-related community-based activities like contact tracing, defaulter tracing, community forums, health promotion, and treatment support. One study participant described their gap as follows:

*“…. I understood that people in the community were unaware of MDR-TB. The majority of health extension workers do not prioritize raising community awareness of MDR-TB”* (FGD-P13). *This was supported by idea from* a district disease prevention head and stated as:

“…. *There is no active system for contacts tracing. Health educators send us information if they find suspected cases. However, some patients might not show up as expected. We have data on three family members who tested positive for MDR-TB”* (KII-P3).

##### Support from a health system

The prime focus of this subtheme is on the enabling elements that DOTS providers require assistance from the current healthcare system for better engagement. All study participants expressed at least two needs to be met from the health system in order for them to effectively participate in MDR-TB prevention, treatment, and management. All study participants agreed that issues with the health system had a negative impact on their engagement in the prevention, treatment, diagnosis, and management of MDR-TB in almost all healthcare facilities. Poor conditions in infrastructure, resources (supplies, equipment, guidelines, and other logistics), capacity building (training), supportive supervision, establishment of public-private partnerships, and assignment of motivated and trained health professionals are some of the barriers that needs to be worked out in order to make them engage better. One of the participants pronounces supplies and logistics problems as:

*“…. The health center I worked in is listed as a DOTS provider. However, it lacks constant electricity, a working microscope, lab supplies, medications, etc, and we refer suspected cases to nearby health centers or district hospitals for AFB-examination and, “Sometimes we use a single kit for many patients and wait for the medication supply for three or more weeks and patients stops a course of therapy that might induce drug resistance”* (FGD-PI04) which was augmented by statement from FGD participant who works at a treatment initiation center:

*“…. We faced critical shortage of supplies and hospital administrators don’t care about funding essential supplies for patient care. For instance, this hospital (the hospital in which this FGD was conducted) can easily handle N-95 masks. Why then they (hospital administrators working in some TIC) can’t do it?”* (FGD-P18).”

Regarding in-service training on MDR-TB, almost all participants pointed out shortage of on-job training mechanisms. One of our FGD participants said:

“….*I missed the new training on MDRTB programmatic management guidelines. I’ve heard that new updates are available. I still work using the old standard”* (FGD-PI05). A health professional working in private clinic heightens the severity of training shortage as:

*“…. We have not participated in TB/MDR-TB guidelines training. You know, most of for-profit healthcare facilities do not provide any training for their staff. I’m not sure if I’m following the (TB/MDR-TB) guideline”* (FGD-P14). One of our key informant interview participants; MDR-TB center focal person suggested the need for training as:

*“…. I’ve received training on the MDR-TB services and public-private partnership strategy. It was crucial in my opinion for better engagement. It is provided for our staff [MDRTB center focal person]. However, this has not yet been expanded to other health facilities”* (KII-P04).

Concerning infrastructures, transportation problem was one of the frequently mentioned obstacles by many participants that hinder engagement in MDR-TB/TB service. This factor had a negative impact to both sides (health professionals and patients). One of discussants said:

*“…. I face obstacles such as transport cost to perform effective TB/MDR-TB outreach activities like health education, tracing family contacts and defaulters and community mobilization. Rural kebeles are far apart from each other. How can I support 6 rural Kebeles?”* (FGD-P01). One of the participants; MDR-TB treatment centers supervisor/program partner seconded the above idea as:

*“…. I suggest government must establish a system to support health professionals working in remote health care facilities in addition to MDR-TB centers. I guess there are more than 30 government health centers and additional private clinics. We can’t reach them all due to transportation challenges”* (KII-P05). *One of the participants*, a district disease prevention head added:

“…. *Our laboratory technicians take sample from MDR-TB suspects to the post office and then, the post office sends to MDR-TB site. Sometimes, feedback may not reach timely. There is no any system to cover transportation cost. That would make case detection challenging”* (FGD-P02).

##### Support from colleagues

Study participants stated the importance of having coworker with whom they could interconnect. However, eight participants reported that they were discriminated by their workmates for various reasons, such as their perceived fear of exposure to infection and their perception as if health professionals working in TB/MDR-TB unit get more training opportunities and other incentives. One of the focus group discussants said:

“…. *My colleagues [health professional working out of MDR-TB TICs] stigmatize us only due to our work assignment in MDR-TB clinic. I remember that one of my friends who borrowed my headscarf preferred to throw it through a window than handing-over it back safely. Look, how much other health professionals are scared of working in MDR-TB unit. This makes me very upset. I am asking myself that why have I received such training on MDR-TB?”* (FGD-P04).

Some of the participants also perceived that, health professionals working in MDR-TB/TB unit are the only responsible experts regarding MDR-TB care and treatment. Because, other health professionals consider training as if it is an incentive to work in such units. One of the FGD discussants described:

*“… Health professionals who work in other service units are not volunteer to provide DOTS if TB focal person/previously trained staffs are not available. Patients wait for longer time”* (FGD-P11).

#### Health facilities’ poor linkage

This theme demonstrates how various healthcare facilities, including private and public healthcare facilities such as, health posts, health care centers and hospitals, and healthcare professionals working at various levels of the healthcare system in relation to TB/MDR-TB service, are inter-linked or communicating with one another.

Many study participants noted a lack of coordination between higher referral hospitals, TB clinics, health posts, and health centers. Additionally, the majority of the assigned healthcare professionals had trouble communicating with patients and their coworkers. A focus group discussant also supported this idea as

*“…. There is a lack of communication between us [DOTS providers at treatment initiation centers] and health posts, health centers, and private clinics. We are expected to support about 30 public health facilities. It’s of too much number, you know. They are out of our reach. We only took action when a problem arose”* (FGD-P16).

Significant number of participants had raised the problem of poor communication between health facilities and treatment initiation centers. One of the interviewees [program manager] said:

*“…. I see that one of our challenges is the weak referral connections between treatment initiation centers and health centers. As a result, improper sample transfer to Gene- Xpert sites and irregular postal delivery are frequent”*. *“Our; DOTS staff at the MDR-TB center, DOTS staff at the health center, and health extension workers are not well connected to one another. Many patients I encountered came to this center [MDR-TB center] after bypassing both health post and health center. Poor linkage and communication, in my opinion, could be one of the causes. The same holds true for medical facilities that are both public and private*” (KII-P02).

## Discussion

Engagement of individual healthcare providers is one of the peculiar interventions to achieve the goal of universal access to drug resistance tuberculosis care and services [17]. Healthcare providers engagement in detecting cases, treating and caring for multidrug resistant tuberculosis (MDR-TB) may be influenced by various intrinsic (individual provider factors) and extrinsic (peer, health system, political and other factors) [15]. Our study explored engagement of individual DOTS providers and factors that influence their engagement in MDR-TB prevention and management service. This is addressed through five emergent themes and subthemes as clearly specified in our results section.

The findings showed patients’ socioeconomic constraints were important challenges that influence health professionals’ engagement, and provision of MDR-TB prevention and management services. Although approaches differ, studies in Ethiopia [24], South Africa [25] and India [26, 27] documented that such factors influence health providers’ engagement in the prevention and management of multi drug resistant tuberculosis. Again, the alleviation of these factors demands the effort from patients, stakeholders working on TB, others sectors, and the healthcare system so that healthcare providers can deliver the service more effectively in their day-to-day activities and will be more receptive to the other key factors.

We explored participants’ experiences on how patients’ awareness about drug sensitive or multi drug resistant tuberculosis influenced their engagement. Accordingly, participants encountered numerous gaps that restricted their interactions with TB/MDR-TB patients. In fact, our study design and purposes vary, studies [28–30] indicated that patients awareness influenced providers decision in relation to MDR-TB services and patients’ awareness status is among factors influencing healthcare providers’ decision making about the care the MDR-TB patient receives. As to our knowledge, patients’ perceived fear of discrimination was not documented whether it had direct negative impact on reducing providers’ engagement. Therefore, patients’ awareness creation is an important responsibility that needs to be addressed by the community health development army, health extension workers, all other healthcare providers and stakeholder for better MDR-TB services and patient outcomes.

Our study indicates that healthcare providers perceived that they would be exposed to MDR-TB while they are engaged. Some of the participants were more concerned about the disadvantages of engagement in providing care to MDR-TB patients which were predominantly psychological and physical pressure. In this context, the participants emphasized that engagement in MDR-TB patient care is “always being at risk” and expressed a negative attitude. This finding is similar to what has been demonstrated in a cross-sectional study conducted in South Africa in which majority of healthcare providers believed their engagement in MDR-TB services would risk their health [21].

However, majority of the healthcare providers demonstrated perceived fear of exposures mainly due to poor infection prevention practices and substandard organization of work environment in most TB/MDR-TB units. This is essentially reasonable fear, and needs urgent intervention to protect healthcare providers from worsening/reducing their effective engagement in MDR-TB patient care. On the other side of the coin, perceived risk of occupational exposure to infection could be source for taking care of oneself to combat the spread of the infection.

In our study, healthcare provider’s capability (competence) also had an impact on their ability to engage in prevention and management of MDR-TB. Here, participants had frequently raised their and other healthcare providers’ experience regarding skill gaps, negative attitude towards the service unit they were working in, ineffective use of MDR-TB guideline, poor infection prevention practices and commitment. In addition, many health professionals report serious problems regarding case identification and screening, drug administration, and side effect management. These findings were supported by other studies in Ethiopia [7] and in Nigeria [19, 20]. This implies an urgent need for training of health care worker on how to engage in prevention and management of multidrug resistant TB.

Moreover, our findings provide insights into the role of community TB prevention actors, currently functioning health system, and colleagues and other stakeholders’ regarding healthcare providers’ engagement. Participants emphasized that support from community TB prevention actors is a key motivation to effectively engage on management and prevention roles towards MDR-TB. Evidence shows that community TB prevention is one of the prominent interventions that study participants would expect in DOTS provision as community is the closest source of information regarding the patients [31, 32].

Similarly, all participants had pointed out the importance of support from a health system directly or indirectly influence their engagement in the prevention, diagnosis, treatment, and management of MDR-TB. Researches indicated that health system supports are enabling factors for healthcare providers in decision making regarding TB/MDR-TB prevention and treatment [33]. This problem is documented by the study done in Ethiopia [22]. In addition, support from colleagues and other stakeholders was also a felt need to engage in MDR-TB which was supported by the World Health Organization guideline which put engagement in preventing MDR-TB and providing patients centered care needs collaborative endeavor among healthcare providers, patients, and other stakeholders [17].

Participants showed that there were poor linkage among/within DOTS providers working in health post (extension workers), health centers, hospitals and MDRTB treatment initiation centers. Our finding is consistent with a research in South Africa which shows poor health care attitude is linked to poor treatment adherence [34]. Our study implies the need for further familiarization especially on clinical programmatic management of drug resistant tuberculosis. Moreover, program managers need to follow health professionals’ engagement approaches recommended by the World Health Organization: End TB strategy [17].

### Limitations of the study

There are some limitations that must be explicitly acknowledged. First, participants from private health facilities were very few, which might have restricted the acquisition and incorporation of perspectives from health care providers from private health care facilities. Second, healthcare providers’ engagement was not measured from patient side given that factors for engagement may differ from what has been said by the healthcare provides. Third, power relationships especially among focus group discussant in MDR-TB treatment initiation centers might have influenced open disclosures of some sensitive issues.

## Conclusion

The study showed how healthcare provider’s engagement in MDR-TB management and prevention was influenced. Accordingly, patient’s underlying causes, seeking support, perceived occupational exposure, healthcare provider’s incompetence and health facilities poor linkage were identified from the analysis. Weak community TB prevention efforts, poor health system support and support from colleagues, health care providers’ incompetence and health facilities poor linkage were among identified factors influencing engagement in MDR – TB prevention and management. Therefore, measures need to be in place that avert the observed obstacles to health professionals’ engagement including further quantitative studies to determine the effects of the identified reasons and potential factors in their engagement status.

Furthermore, our findings pointed out the need for additional training of service providers, particularly in clinical programmatic management of drug-resistant tuberculosis. Besides, program managers must adhere to the World Health Organization’s recommendations for health professional engagement. Higher officials in the health sector needs to strengthen the linkage between health facilities and service providers at different levels. Community awareness creation to avoid fear of discrimination including provision of support for those with MDR-TB is expected from health experts through implementation of social behavioral change communication activities.

### Electronic supplementary material

Below is the link to the electronic supplementary material.

Supplementary Material 1

## Declarations

All methods and report contents were performed in accordance with the standards for reporting qualitative research.

## Abbreviations

DOTS: Directly observed treatment short-course
DS-TB: Drug susceptible tuberculosis
MDGs: Millennium development goals
MDR–TB: Multidrug resistant tuberculosis
SDG: Sustainable development goals
TB: Tuberculosis
TIC: Treatment initiation center
WHO: World Health Organization
XDR–TB: Extensively drug resistant TB
