# Supplementary material for: Exploring health care providers’ engagement in prevention and management of multidrug resistant Tuberculosis and its factors in Hadiya Zone health care facilities: qualitative study
Source: BMC Health Serv Res. 2024 Apr 27;24:542. doi: 10.1186/s12913-024-10911-6 (PMC11056065; doi:10.1186/s12913-024-10911-6)
Supplement: Supplementary file 1 — Supplementary Material 1 [file 12913_2024_10911_MOESM1_ESM.docx]

# Annex-1-English Version Guide for Focus Group Discussion

**Group 1: Guide for focus group discussion of MDR-TB-TICs participants**

| **Key identifying information** |
| --- |
| Location:______________________________________________________________________ |
| Date: _______________________________________________________________________ |
| Time discussion started: ___________________________Time ended: ________________ |
| Participants: total: ________________women: _______________________ |
| **Introduction of Facilitators and FGD Process** |
| - The moderator introduces her(him)self and the note-taker - Explains the purpose of the FGD - Explains ground rules and format of the focus group discussion: Use of tape-recorder; Everyone should participate freely; Everything said by anyone will be kept confidential and anonymous; No right or wrong answers; One person talks at a time; Honest responses are highly appreciated - Please, discuss each theme until saturation of ideas are reached |
| 1. **Introduction of Participants** |
| 1. Ask participants to specify their age, marital status, profession, educational level, length of residence in current health facilities, previous experience including MDR-TB services |
| 1. Ask a nickname that they would like to be called during the discussion |
| 1. Any questions before we start? |
| 1. **Beliefs about MDR-TB in particular** |
| 1. In MDR-TB prevention and control strategies, how do you believe the contribution of preventing susceptible TB to MDR-TB prevention? That is, what does it mean the prevention of susceptible TB to MDR-TB prevention and control? |
| 1. Do you think that MDR-TB could have been emerged out due to failure to implement national tuberculosis guidelines [**PROBE**: health worker compliance to guideline, risk factors other than identified in the TB guideline or other scientific evidences, …? ] |
| 1. **Knowledge about and attitude towards MDR-TB prevention and control** |
| 1. Would you tell us about MDR-TB (**Probe:** definition, cause, transmission, |
| 1. Would you tell about risk factors, prevention, |
| 1. Would you tell us about clinical manifestation? |
| 1. Would you tell us about diagnostic approaches of MDR- TB? |
| 1. Would you please list drugs according to current national treatment guideline for MDR- TB treatment options (**Probe : to** list all drugs correctly**)** |
| 1. Would you tell us MDR-TB treatment regimens according to current national treatment guideline of Ethiopia? (Probe**:** drug combination; duration of treatment, dosage for intensive as well as continuation phases...) |
| 1. Tell us about the experiences of your colleagues with regard to the general knowledge, attitude, and compliance to treatment guideline? |
| 1. Would you or your colleagues like to be assigned in DOTs service in your facility (**Probe:** interest, patient approach, perceived susceptibility, severity of MDR-TB…) |
| 1. What infection prevention activities are being implemented in your locality regarding TB (including MDR-TB)?(probe: screening, health education- issues addressed during HE) |
| 1. What health system factors are being implemented to improve treatment success rate of MDR-TB treatment? (Probe: financial, motivational, supervision, training, intersectoral collaboration, patient support including their family, policies….. |
| 1. What further action would you advice in order to achieving good treatment outcomes for patients with MDR-TB and to prevent and control it? |
| 1. How do you see occupational risks or hazards of MDRTB (probe perceived hazards) |
| 1. How do you see perceived origin or emergence of MDRTB? |

**Group 2: Guide for Focus Group Discussions of DOTs providers**

| **Key identifying information** |
| --- |
| Location:______________________________________________________________________ |
| Date: _______________________________________________________________________ |
| Time discussion started: ___________________________Time ended: ________________ |
| Participants: total: ________________women: _______________________ |
| **Introduction of Facilitators and FGD Process** |
| - The moderator introduces her(him)self and the note-taker - Explains the purpose of the FGD - Explains ground rules and format of the focus group discussion: Use of tape-recorder; Everyone should participate freely; Everything said by anyone will be kept confidential and anonymous; No right or wrong answers; One person talks at a time; Honest responses are highly appreciated - Please, discuss each theme until saturation of ideas are reached |
| 1. **Introduction of Participants** |
| 1. Ask participants to specify their age, marital status, profession, educational level, length of residence in current health facilities, previous experience including MDR-TB services, |
| 1. Ask a nickname that they would like to be called during the discussion |
| 1. Any questions before we start? |
| 1. **Beliefs about MDR-TB in particular** |
| 1. In MDR-TB prevention and control strategies, how do you believe the contribution of preventing susceptible TB to MDR-TB prevention ? That is, what does it mean the prevention of susceptible TB to MDR-TB prevention and control? |
| 1. Do you think that MDR-TB could have been emerged out of the failure to implement national tuberculosis guidelines [**PROBE**: health worker compliance to guideline, risk factors other than identified in the TB guideline or other scientific evidences, …? ] |
| 1. **Knowledge about and attitude towards MDR-TB prevention and control** |
| 1. Would you tell us about MDR-TB (**Probe:** definition, cause, transmission, |
| 1. Would you tell about risk factors, prevention, |
| 1. What are your criteria to suspect a person as having MDR-TB? |
| 1. Would you tell us about diagnostic approaches of MDR- TB? |
| 1. Would you please list drugs according to current national treatment guideline for MDR- TB treatment options (**Probe : to** list all drugs correctly**)** |
| 1. Would you tell us MDR-TB treatment regimens according to current national treatment guideline of Ethiopia? (Probe**:** drug combination; duration of treatment, dosage for intensive as well as continuation phases...) |
| 1. Tell us your experiences about your colleagues with regard to the general knowledge, attitude, and compliance to treatment guideline? |
| 1. Would you or your colleagues like to be assigned in DOTs service in your facility (**Probe:** interest, patient approach, perceived susceptibility, severity of MDR-TB…) |
| 1. What does mycobacterium complex mean**?** |
| 1. Would you tell us about TB (probe: definition, causes, transmission**….** |
| 1. Would you tell about risk factors, prevention |
| 1. Would you tell us about clinical manifestation? |
| 1. Would you tell us about diagnostic approaches of **DS- TB**? |
| 1. Would you please list drugs according to current national treatment guideline for **DS- TB**  treatment options (**Probe : to** list all drugs correctly**)** |
| 1. Would you tell us **DS-TB** treatment regimens according to current national treatment guideline of Ethiopia? (Probe**:** drug combination; duration of treatment, dosage for intensive as well as continuation phases...) |
| 1. **Community activities** |
| 1. Would you tell us your experience regarding MDR-TB in your community (suspected or confirmed)? |
| 1. Have you traced close contacts of MDR-TB patients (suspected or confirmed)? If any….. |
| 1. What activities are being implemented in your locality regarding TB (including MDR-TB)? (probe: screening, health education- issues addressed during HE, tracing loss to follow up ) |
| 1. **Health system factors** |
| 1. What health system factors are being implemented to improve success rate of MDR-TB treatment? (Probe: financial, motivational, training, intersectoral collaboration, patient support including their family, policies, linkage with private health facilities like clinic, private hospital….. |
| 1. Identify and supervise treatment supporters (**Probe: HEW, HDA, Community forum….** |
| 1. What further action would you advice in order to achieving good treatment results for patients with MDR-TB and to prevent and control it? |
| 1. How do you see occupational risks or hazards of MDRTB (probe perceived hazards) |
| 1. How do you see perceived origin or emergence of MDRTB? |

# English Version Guides for Key informants and in-depth interview

**General information**

1. Name of the woreda _______________________________________________________
2. Number of TB treatment centers in the woreda__________________________________
3. Number of MDR-TB treatment centers in the woreda____________________________
4. Number of MDRTB cases enrolled at woreda level______________
5. Number of DSTB cases enrolled at woreda level_______________________________
6. Number of professionals trained on new national TB treatment guidelines______
7. Number of professionals trained on PMDRTB____________
8. What MDRTB preventive measures are currently in practice (probe: approach, community activities, health system activities,

**II. Health system factors**

- 1. What health system factors are being implemented to improve treatment success rate of DSTB and MDR-TB treatment? (Probe: financial, motivational, training, patient and community education, intersectoral collaboration, patient support including their family, policies, linkage of private health facilities like (clinic, private hospital) and public health facilities with TIC …..
  2. Social support made to patients at community level (probe: material, financial, food etc
  3. Identify and supervise treatment supporters (Probe: HEW, HDA, Community forum….
  4. Treatment supplies and accessibility (drugs, guidelines, PPE, reagents
  5. Quality assurance for laboratories
  6. Community exposure prevention for MDRTB (probe: patient movement outside TIC, PPE for patients, patient family contact, workplace contact, case transportation to treatment follow up and vice versa
  7. Health information management regarding TB (probe: tracing mechanisms, early case detection and notification, reporting challenges …
  8. How do you summarize major challenges in the prevention and control of MDR-TB?
  9. What further action would you advice in order to achieving good treatment results for patients with MDR-TB and to prevent and control it? (opportunities
  10. Knowledge, skills and attitude of health care professionals towards the prevention and control of DSTB and MDRTB? (probe: transfer of learning, motivation and willingness to participate in the prevention and control efforts of MDRTB,
  11. Treatment adherence challenges in both DSTB and MDRTB management
  12. Supportive supervision made by higher health personnel regarding TBc in general
  13. Critical control points (probe: schools, prisons, transportation, police stations, health facilities, gatherings)
  14. How do you describe the adequacy of trained personnel in the management of MDRTB?
  15. How do you describe special financial support (regular budget, donors) for MDRTB prevention and control?
  16. What is your experiences regarding the existing management procedures of MDRTB at private sector? (Probe: treatment regimen, guideline compliance, patient support, supplies, diagnosis and treatment challenges, decision procedures when new cases of MDRTB emerge etc.)
  17. Mix of private and public approach of TB care (Probe: source of care, volunteer public, volunteer private)
  18. Control mechanisms of TB drugs on the market

**English version of observation and document review checklist**

**Name of the health facility___________________________________________**

1. Location appropriateness of the TBC room 1. Appropriate Not appropriate
2. Training status of the TBC personnel 1. Yes 2. No
3. Presence of TBC management guideline in the center 1. Yes 2. No
4. Follow up and defaulter tracing mechanism (2yeares period) for MDR-Tbc 1.Yes 2.No
5. Follow up and defaulter tracing mechanism (2yeares period) for DS-Tbc 1.Yes 2.No
6. Ventilation system of the room 1. Ventilated 2. Poorly ventilated 3.Not ventilated
7. Availability of PPE like respirators 1. Yes 2. No
8. Use of the PPE 1. Yes 2. No
9. Room equipped with necessary materials (TB register, ventilator, Respirators, etc) 1. Yes 2. No
10. Seat arrangements of the treatment room 1. Designed to assist prevention 2. Not
11. Laboratory setup (supplies and equipment for the prevention and control) 1. Adequate 2. Not adequate
12. Accessibility and linkage to GeneXpert sites
